# 3D Self‐Supported Visible Light Photochemical Nanocatalysts

**DOI:** 10.1002/advs.202502981

**Published:** 2025-03-24

**Authors:** Fateh Mikaeili, Mohammad Mahafuzur Rahaman, Pelagia‐Irene (Perena) Gouma

**Affiliations:** ^1^ Department of Materials Science and Engineering The Ohio State University 140 W. 19th Avenue Columbus OH 43210 USA; ^2^ Department of Mechanical and Aerospace Engineering The Ohio State University 201 W. 19th Avenue Columbus OH 43210 USA

**Keywords:** Cu‐doped TiO_2_, nanofibers, self‐supported mats, transmission electron microscopy, visible light photocatalyst

## Abstract

This work focuses on 3D, self‐supported, nanofibrous TiO_2_ structures (nanogrids) prepared using blend electrospinning. The presence of anatase and brookite phases in Cu‐doped TiO_2_ nanogrids significantly enhances the photocatalytic properties of the titania system. The absorption edge in Cu‐doped TiO_2_ shifts to the visible due to the narrowed bandgap and efficient separation of photogenerated charge carriers facilitated by Cu doping. The presence of the brookite phase further contributes to the enhanced performance, by reducing electron–hole recombination. A wide range of characterization techniques, including cyclic voltammetry and chronoamperometry studies which show that the Cu doped TiO₂ sample generates a significant photocurrent under visible light, are employed to elucidate the role of Cu doping in enhancing the visible light photocatalytic efficiency of TiO_2_ nanogrids, offering valuable insights for developing advanced photochemical catalysts for environmental and energy applications. The nanogrids studied here are far superior to P25 Degussa and are activated by natural sunlight and do not require a filtration system to remove nanoparticles from the water. These self‐supported nanofibrous photochemical catalysts offer all the benefits of nanomaterials while suffering from none of their drawbacks.

## Graphical abstract

This study presents 3D, self‐supported TiO_2_ nanogrids with enhanced visible‐light photocatalytic efficiency due to Cu doping and the presence of anatase and brookite phases. These nanogrids outperform P25 Degussa, operate under natural sunlight, and eliminate nanoparticle filtration, making them highly effective for environmental and energy applications.

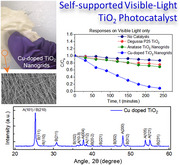

## 1 Introduction

Titanium dioxide (TiO₂) is a widely used functional oxide with a broad range of applications including solar cells, gas sensors, photocatalysis, and self‐cleaning technologies.^[^ ^1^ ^]^ As highlighted in the review on ideal photocatalysts by McKone et al., the development of solar fuel systems faces significant constraints related to the efficiency, stability, and scalability of their photocatalysts.^[^ ^2^ ^]^ The bandgap of TiO₂ (≈3.2 eV) requires excitation by ultraviolet light (below 400 nm), rendering visible light unusable to create electron–hole pairs.^[^ ^3^ ^]^ Second, the short lifetime of the photogenerated electrons and holes in TiO₂ nanoparticles hinders their effective utilization.^[^ ^4^ ^]^ Finally, the lack of an ideal self‐supported nanostructure that can be easily recovered remains a challenge.^[^ ^5^ ^]^

To overcome these limitations and enhance the photocatalytic properties of TiO₂ under visible light, numerous studies have been explored doping with various metal ions such as Ag, Fe, Pd, Pt, Zn, Mn, B, and Zr.^[^ ^6^ ^]^ Cu doping has shown promise;^[^ ^7^ ^]^ however, it is required to reduce the optical bandgap further and to increase the surface area to volume ratio of Cu‐doped titania to achieve the desirable photocatalytic activity.^[^ ^8^ ^]^ Incorporation of Cu into the lattice of TiO_2_ though leads to the formation of unwanted CuO and Cu₂O by‐products.^[^ ^9^ ^]^ Copper oxide systems, Cu₂O and CuO, are abundant, nontoxic, natural p‐type semiconductors with bandgaps (≈2.1 eV for Cu₂O and ≈1.2 eV for CuO) suitable for photoinduced water splitting under visible light.^[^ ^10^ ^]^ However, these materials exhibit instability in electrolytic solutions due to their tendency to undergo photooxidation. Siripala et al. prepared a Cu₂O/TiO₂ heterojunction by electrodeposition of copper oxide, but the combination of TiO₂ and CuO crystallites proved problematic due to inconsistent band bending, varying crystallite sizes, and the issue of CuO crystallite sulfidation.^[^ ^11^ ^]^ Therefore, designing a system that incorporates Cu ions as intercalants or substitutions in the TiO₂ lattice is important.

This work offers a viable route to obtain stable Cu‐doped TiO_2_ nanoparticles with a high surface area‐to‐volume ratio by means of nanofiber‐based processes.

Electrospinning is a commonly used technique for preparing long, continuous nanofibers.^[^ ^8b^ ^]^ While there are several reports on rare‐earth‐doped TiO₂ nanofiber structures in the literature,^[^ ^12^ ^]^ synthesizing self‐supported 3D mats of Cu‐doped TiO₂ nanofibers is seldom reported. TiO₂ nanomats doped with Cu (called nanogrids) are studied here on how they alter the optical and electronic properties of the titania system.

## 2 Results and Discussion

**Figure 1:**
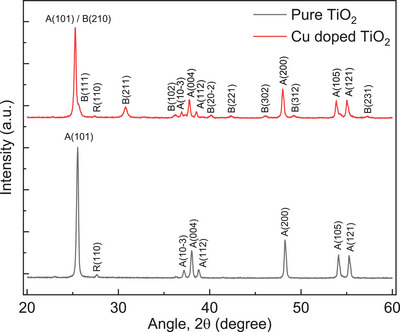
XRD spectra of Pure and Cu‐doped TiO_2_ nanofibers heat‐treated at 500°C [the phases are identified using the PDF file no. 98‐000‐0081, 98‐000‐0128, and 98‐000‐0375 for anatase (A), brookite (B), and rutile (R) respectively].

The undoped and Cu‐doped TiO_2_ nanofibrous mats were analyzed using the XRD method to confirm their crystallinity and phase composition after the heat treatment process, as shown in **Figure** **1**. The analysis reveals that both samples consist of anatase phase with a prominent peak of (101) plane, with almost no rutile formation.^[^ ^13^ ^]^ The anatase (101) peak shifted toward a lower diffraction angle for 2 at% Cu‐doped TiO_2_ compared to pristine TiO_2_. The decrease of this peak is due to the presence of brookite (210) peak overlapping with the (101) of anatase due to the Cu‐ doping of TiO_2_. The notable shift in the diffracting angle and absence of any peaks for pure Cu or Cu‐(I or II) oxides indicate the successful incorporation of Cu into the TiO_2_ lattice, a crucial signature for ensuring the doping of Cu in the TiO₂ lattice. The presence of the metastable brookite phase alongside anatase is also confirmed by the characteristic (211), (102), (202), (221), (302), (312), (231) peaks at 30.89°_,_ 36.28°, 40.2°, 42.33°, 46.1°, 49.2°, 57.4° and a shoulder peak of plane (111) at 25.7° originated near the anatase (101) peak.^[^ ^14^ ^]^ Brookite is a desirable polymorph due to its unique electron trap depth, which prolongs electron–hole pairing and maintains high energy levels necessary for efficient photochemical reactions. This combination of phases enhances the photocatalytic properties of the material, making it suitable for advanced applications.^[^ ^15^ ^]^

**Figure 2:**
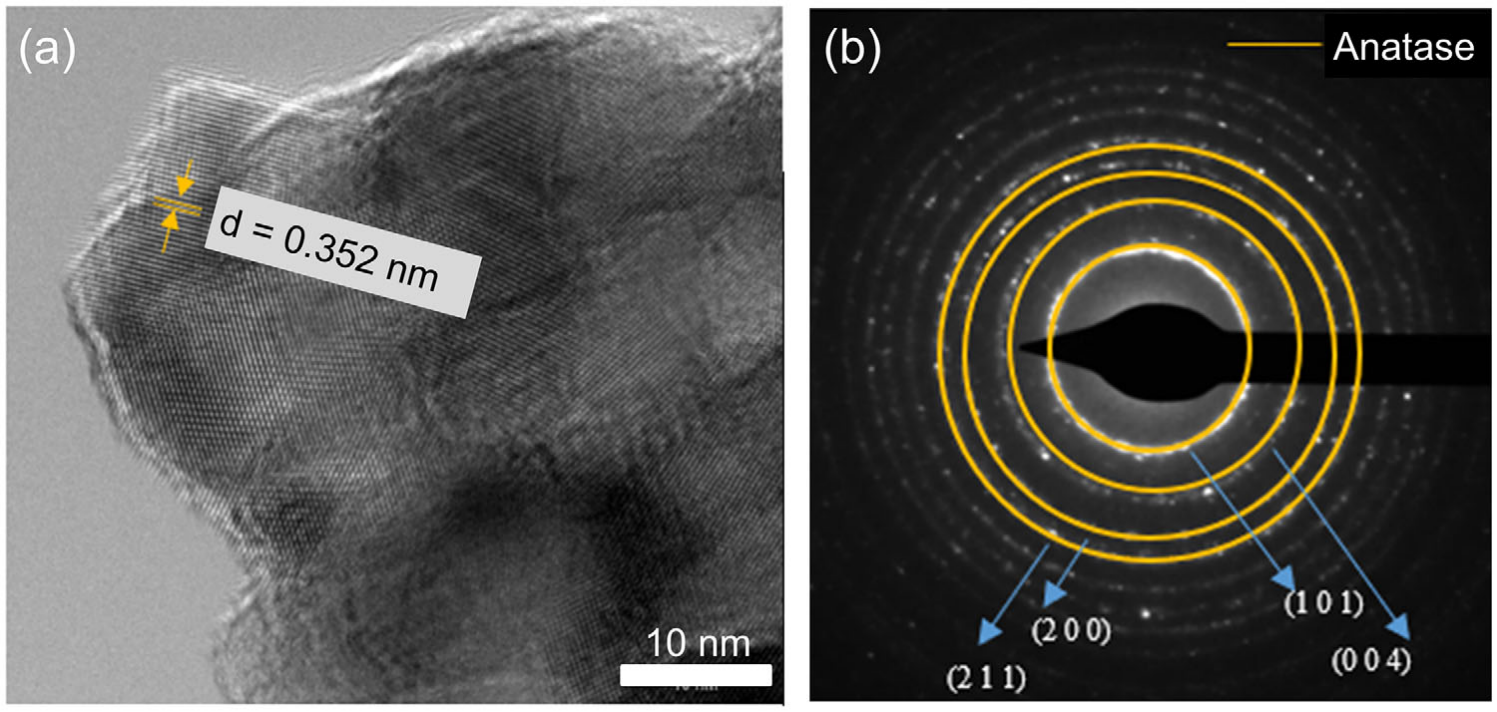
(a) High‐resolution TEM Image showing (101) plane of anatase phase of pure TiO_2_ nanofibers; b) the SAED pattern of the nanofibers.

**Figure 3:**
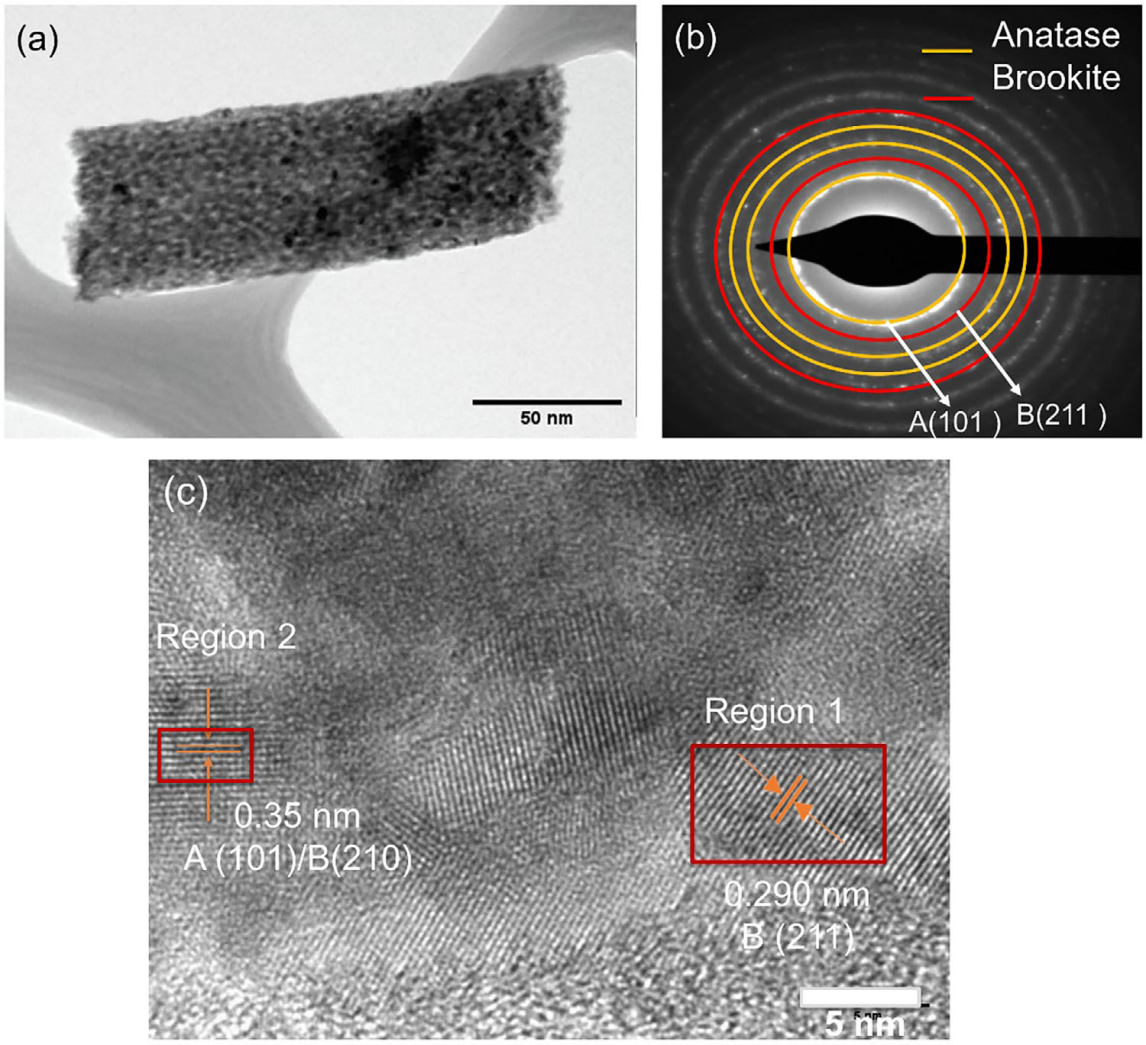
a)TEM image of Cu‐doped TiO_2_ heat‐treated nanofiber b) SAED pattern of Cu‐doped TiO_2_ nanofibers c) high‐resolution TEM depicts crystallites of brookite and anatase phase, respectively.

High‐resolution TEM analysis was conducted on the undoped TiO₂ samples. **Figure** **2** shows the high‐resolution TEM image and the corresponding selected area electron diffraction (SAED) pattern of the undoped TiO₂ nanofibers after heat treatment. The TEM image reveals uniform crystallites of anatase phase TiO₂, with no evidence of brookite or rutile phases. The SAED pattern further confirms the exclusive presence of anatase crystallites, as indicated by the distinct diffraction rings corresponding to the anatase phase. Unlike the doped samples, as shown in **Figure** **3**, no additional phases were observed in the undoped TiO₂, suggesting that it was the introduction of Cu dopants that stabilized the brookite phase. The crystallites of the undoped TiO₂ are evenly distributed and maintain the anatase structure. The absence of other polymorphs in the undoped sample suggests that the observed photocatalytic behavior is solely attributed to the anatase phase, providing a baseline for comparing the effects of Cu doping on the structural and photocatalytic properties of TiO₂. The high‐resolution TEM and SAED analyses collectively confirm that the undoped TiO₂ maintains a stable and pure anatase phase.

High‐resolution TEM and selected area diffraction patterns were obtained to characterize the Cu‐doped samples, as shown in Figure 3. The SAED ring pattern of the doped sample matches the d‐spacing observed in the high‐resolution TEM image, with a d‐spacing of 2.91 nm corresponding to the (2 1 1) plane of the brookite phase, validating the XRD results. Brookite crystallites are uniformly distributed across the nanofibers, in close proximity to anatase crystallites, as shown in the dark field images. This even distribution ensures that the unique properties of brookite are effectively integrated into the overall photocatalytic performance of the material.

**Figure 4:**
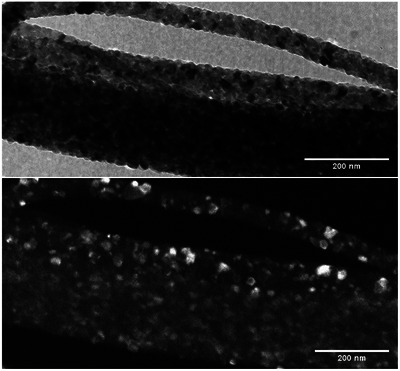
Dark Field image of the brookite phase in the Cu‐Doped TiO_2_ Nanofibers.

To fully map the location of the brookite phase in the doped sample, an objective aperture was used around the ring pattern associated with the brookite phase. By doing so, an image was captured in dark field mode in the TEM, brightening the crystallites of brookite. The results, shown in **Figure** **4**, indicate that brookite crystallites are uniformly distributed around the fiber with no preferential growth direction. Furthermore, the brookite crystallites appear larger in diameter compared to the surrounding anatase crystallites. This observation is consistent with the natural tendency of brookite to form larger crystallites (10–30 nm) compared to anatase (9–15 nm) due to its lower surface energy. The presence of brookite in this size range and distribution profile contributes to the overall increase in photocatalytic efficiency by providing additional active sites for electron–hole separation.

**Figure 5:**
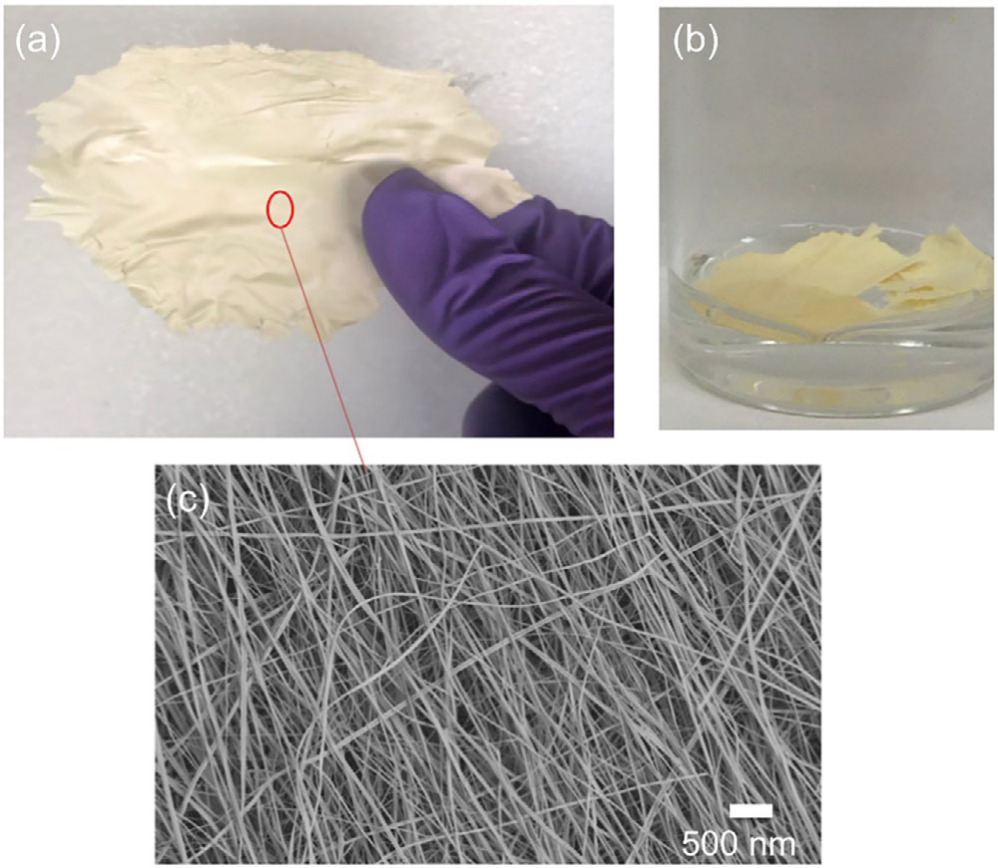
a,b) Cu‐doped TiO_2_ heat‐treated nanofiber blankets, c) SEM image of the nanofiber.

The heat‐treated and doped TiO₂ mats are self‐supported and can be shaped into various forms as photocatalytic mats and blankets, as shown in **Figure** **5**a,b. Their microstructure consists of nonwoven fibers with diameters ranging from 60–190 nm and lengths extending to several microns. This nanofibrous mat floats on water. It is a porous polycrystalline grid of interconnected grains with a texture of potato chips, as shown in Figure 5a. It is very light and robust, making these mats suitable for applications in water remediation or water splitting. The high aspect ratio of the nanofibers is crucial for efficient photocatalysis, as it increases the interface area between light, semiconductor, and solution, thereby enhancing the interaction and efficiency of the photochemical catalytic process. The distinct yellow color of the mats, in contrast to the white color of pure TiO₂ (please see the supplementary Figure S1, Supporting Information), suggests a shift toward visible light absorption, which is indicative of successful Cu doping and its impact on the electronic properties of the material.

**Figure 6:**
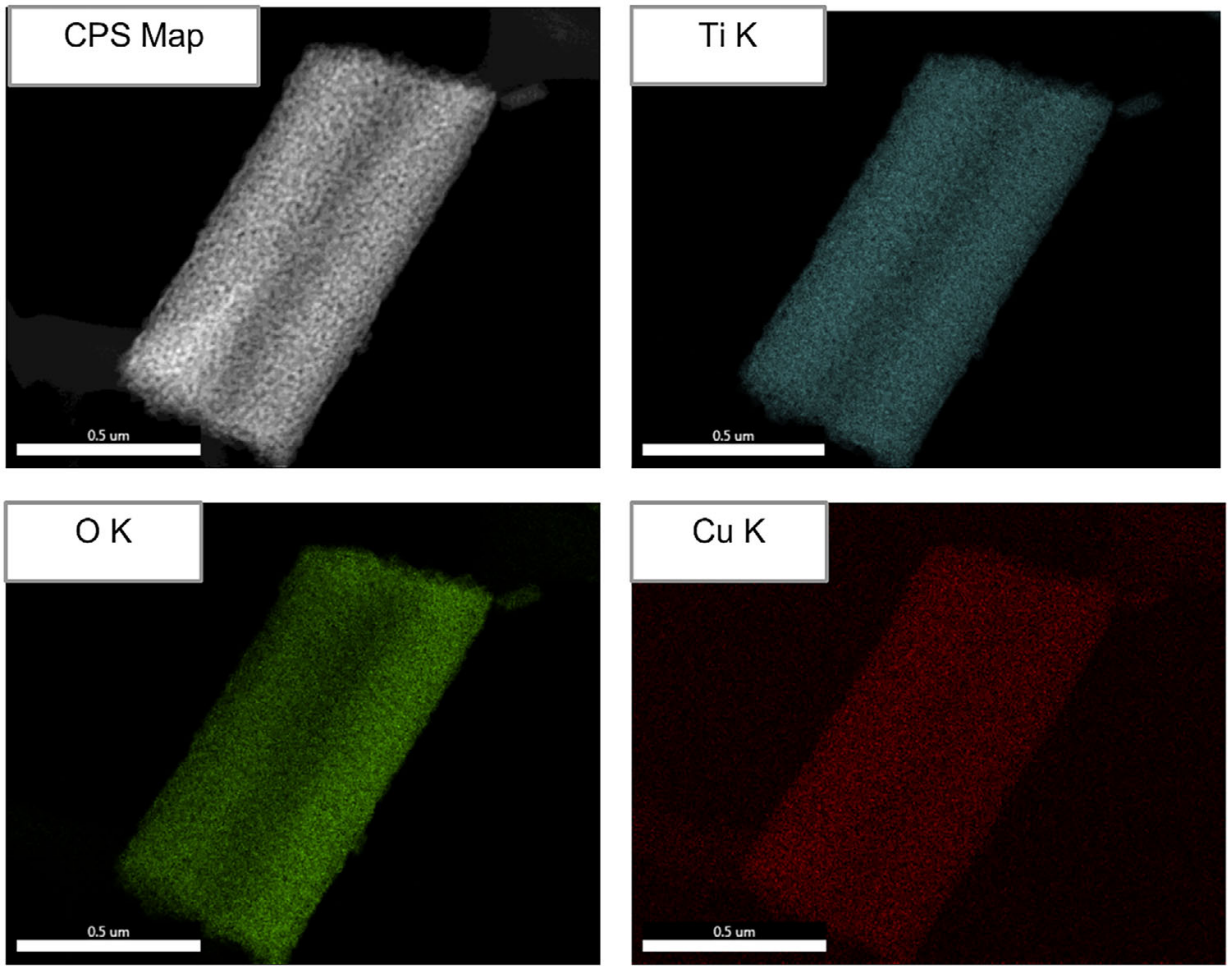
EDX elemental mapping of the Cu Doped TiO_2_ electrospun nanofibers.

To map the distribution of dopants, high magnification EDX mapping and spectral analysis of both heat‐treated and non‐heat‐treated fibers were performed. EDX spectra of supplementary Figure S2 (Supporting Information) indicate the complete removal of carbon from the PVP carrier polymer during heat treatment. This ensures that the final product is free from organic contaminants that could affect its photocatalytic performance. High‐resolution EDX mapping across a single nanofiber in **Figure** **6** shows uniform distribution of Ti, O, and Cu elements, confirming successful doping at the atomic scale without forming CuO, Cu₂O, or other impurities. This uniform distribution is critical for maintaining the electronic and structural integrity of the nanofibers, ensuring consistent and reliable photocatalytic activity across the material.

**Figure 7:**
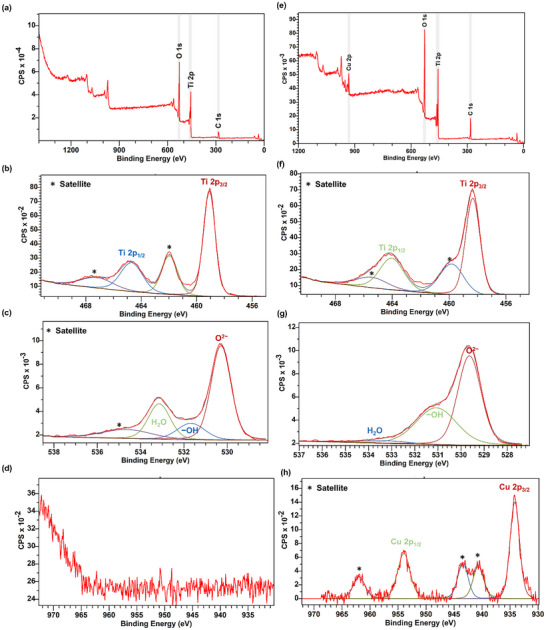
a) Survey XPS spectrum of pure TiO_2_ nanofibrous mat, b–d) High‐resolution deconvoluted XPS spectra of Ti 2p, O 1s, and Cu 2p for pure TiO_2_; e) wide scan XPS survey of Cu‐doped TiO_2_ nanofibrous mats; and f–h) deconvoluted XPS spectra of Ti 2p, O 1s, and Cu 2p for Cu‐doped TiO_2_ nanofibrous mat, respectively.

To fully understand the oxidation states and the chemical environment of the Cu dopant in the TiO_2_ lattice, X‐ray photoelectron spectroscopy (XPS) analysis was conducted on both the doped and undoped TiO_2_ nanofibers. **Figure** **7**a,e presents a wide scan survey containing peaks for Ti, Cu, O, and C of the undoped and doped samples, respectively. Also, a comprehensive analysis is performed for the oxidation states of Ti 2p, O 1s, and Cu 2p lines of XPS spectra for both samples and presented in Figure 7b–d for undoped, and Figure 7f–h for Cu‐doped samples, accordingly. The corresponding peak energies, and full width at half maxima (FWHM) are summarized in **Table** **1**. The data was analyzed in CasaXPS software, where C 1s peak at 284.8 eV was used for calibrating excess charges of the states.^[^ ^16^ ^]^

**Table 1:** XPS results on peak energies, and full‐width half maxima (FWHM) of Ti 2p, O 1s, and Cu 2p lines of pure TiO_2_ and Cu‐doped TiO_2_ nanofiber mats.

Samples	Component		Peak Energy [eV]	FWHM [eV]
TiO_2_	Ti 2p_3/2_		459.09	1.02
	Ti 2p_1/2_		464.71	1.87
		Lattice O^2−^	530.29	1.08
	O1s	Hydroxyl	531.68	1.50
		H_2_O	533.14	1.18
Cu‐doped TiO_2_	Ti 2p_3/2_		458.34	1.19
	Ti 2p_1/2_		464.04	2.00
	Cu 2p_3/2_		934.16	2.01
	Cu 2p_1/2_		953.97	2.67
		Lattice O^2−^	529.64	1.18
	O1s	Hydroxyl	531.07	2.06
		H_2_O	533.31	2.42

The undoped TiO_2_ nanofibers show no Cu 2p signals as observed from Figure 7d, as expected since no copper was introduced into the system. In Figure 7b, the Ti 2p spectrum of the undoped sample reveals peaks at binding energies of 459.09 and 464.71 eV, corresponding to Ti 2p₃/₂ and Ti 2p₁/₂ states, respectively. These peaks slightly shifted toward the lower binding energies at 458.34 and 464.04 eV in the Cu‐doped TiO_2_ nanofiber mat as shown in Figure 7f. These binding energies correspond to the Ti^4+^ oxidation state of TiO_2_ and provide insight into the absence of Ti^3+^ states in the materials.^[^ ^17^ ^]^ Also, the presence of Cu^2+^ state in doped TiO_2_ mat is confirmed by the Cu 2p_3/2_ and Cu 2p_1/2_ peaks at 934.16 and 953.97 eV respectively in Figure 7h, further confirming the absence of pure Cu (Cu^0^‐state) or Cu_2_O (Cu^+^ state).^[^ ^18^ ^]^ In addition, the shift of Ti 2p_3/2_ and Ti 2p_1/2_ peaks toward lower energy can be attributed to the incorporation of Cu^2^⁺ ions into the TiO₂ lattice. The incorporation of Cu into TiO_2_ causes changes in the electronic environment around the Ti^4+^ ions. The shift suggests that Cu doping introduces new electronic states and modifies the local structure, potentially creating oxygen vacancies or altering the bond lengths and angles within the TiO₂ lattice.

The O 1s spectra of undoped TiO_2_ in Figure 7c contains a well‐formed peak at 530.29 eV, and two shoulders at 531.68 and 533.14 eV. The binding energy around 530.29 eV corresponds to the lattice oxygen of TiO_2_ whereas the peak energies at 531.68 and 533.14 eV are attributed to the presence of hydroxyls and physisorbed water on the surface.^[^ ^19^ ^]^ In the Cu‐doped TiO_2_ sample, the lattice oxygen peak of TiO_2_ switches toward lower binding energy at 529.64 eV, presented in Figure 7g. A similar type of shift is also observed in Ti 2p peaks. The downward peak shifts of both Ti 2p and lattice oxygen in the Cu‐doped TiO_2_ sample further confirm that the electronic states and local structure of TiO_2_ lattice are changing from Cu‐doping.

The Cu^0^, Cu^+^ oxidation states, and CuO are absent in the doped sample as confirmed from XPS and XRD spectra respectively, providing insight that Cu is only incorporated into the TiO_2_ lattice as a substitution of Ti. The calculated chemical formula for pure TiO_2_ and Cu‐doped TiO_2_ samples are TiO_1.92_ and Cu_0.08_Ti_0.92_O_1.88_, respectively. This suggests that the Cu^2+^ is substituting Ti^4+^ ions and increasing the oxygen vacancies in the doped sample.

The UV–vis analysis performed on both samples aimed to determine their bandgaps and confirm the visible light photocatalysis capability of the Cu‐doped TiO₂. Figure S3 (Supporting Information) shows the UV–vis spectra in reflectance mode. Pure TiO₂ fibers absorb light at wavelengths of 400 nm or less, while the Cu‐doped TiO₂ exhibits a broader absorption spectrum extending to 500 nm, indicating successful engineering for visible light activity. The Tauc plot in reference^[^ ^5^ ^]^ using the Kubelka–Munk transformation reveals bandgaps of 3.1 eV for pure TiO₂ and 2.62 eV for Cu‐doped TiO₂, confirming the enhanced visible light activity of the doped sample. This shift in bandgap is a direct result of Cu doping, which modifies the electronic structure and allows the material to utilize a broader range of the solar spectrum, thereby improving its photocatalytic efficiency under visible light.

**Figure 8:**
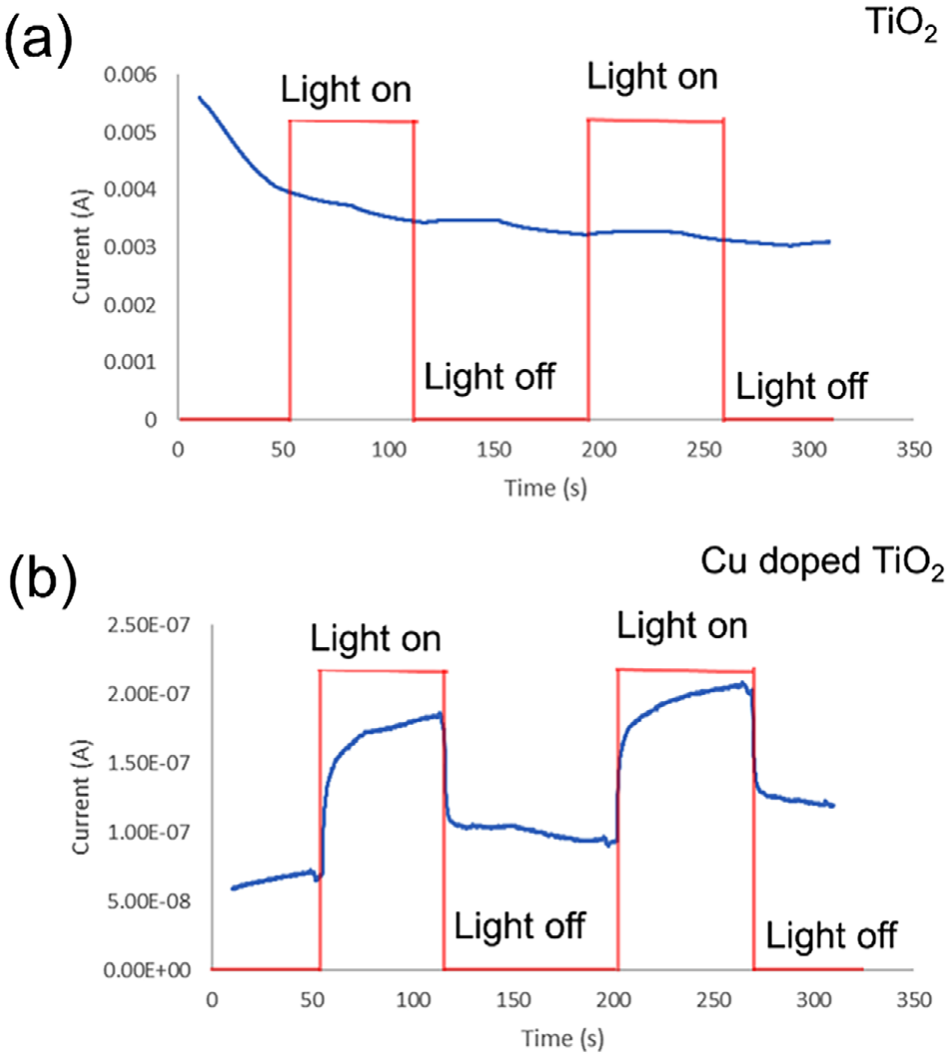
a,b) Chronoamperometry of undoped and Cu‐doped TiO_2_ nanofibers, respectively.

Cyclic voltammetry (CV) was used to establish a baseline during chronoamperometry. The CV curve in 0.1 M KOH solution (Figure S4, Supporting Information). Chronoamperometry in **Figure** **8**a also shows no photocurrent increase under UV‐filtered xenon lamp illumination, as expected due to the TiO₂ bandgap of 3.2 eV. In contrast, the doped sample in Figure 8b shows clear current generation under visible light, with a maximum current of 180 nA, indicating electron–hole pair generation from visible light excitation. This demonstrates the enhanced photocatalytic activity of the doped sample under visible light, confirming the effectiveness of Cu doping in modifying the electronic properties of TiO₂.

**Figure 9:**
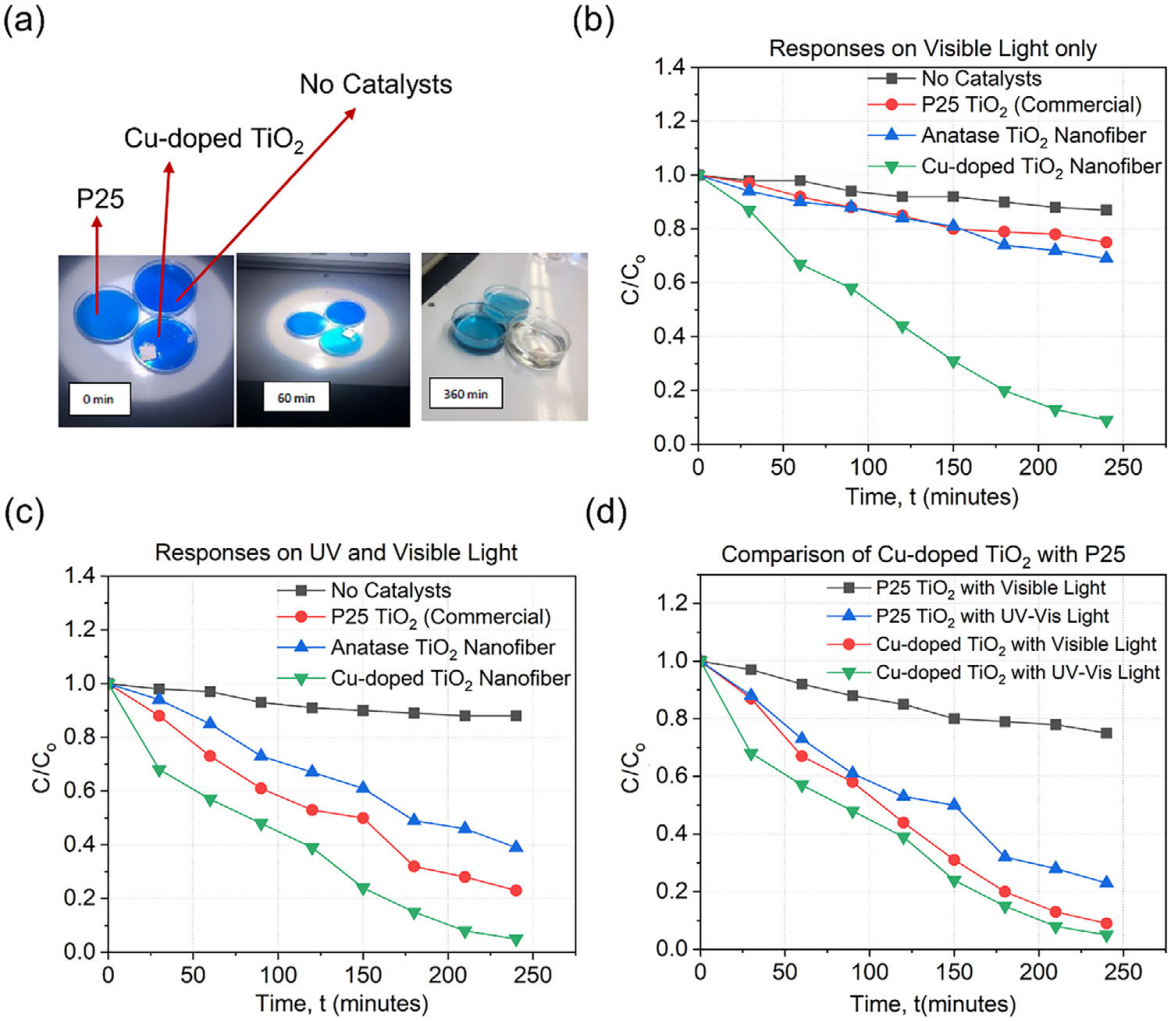
Degradation of Methylene blue. a) Qualitative comparison between the Cu‐doped TiO_2_ and the commercial P25 Degussa TiO_2_ and a container with no photocatalyst; b) UV Filter On and c) UV Filter Off −240 min total Comparing mixed anatase and brookite of Cu‐doped TiO_2_, anatase of undoped TiO_2_ and P25 Degussa powder; d) Visible light versus Full Spectrum comparison of Cu‐doped TiO_2_ with P25 powder.

The photocatalytic activity of Cu‐doped TiO₂ was qualitatively compared with commercial P25 TiO₂. The experiment was performed using a control sample in a petri dish containing 10 mL of 50 ppm Methylene Blue (MB) as shown in **Figure** **9**a. A UV‐filtered xenon lamp was used to block wavelengths below 400 nm. The P25 sample showed minimal degradation, while the doped sample fully degraded Methylene Blue over 360 min, as captured in the provided time‐lapse video. This qualitative analysis highlights the superior performance of the Cu‐doped sample under visible light conditions, emphasizing the impact of Cu doping on the photocatalytic efficiency of TiO₂.

To quantitatively assess the photocatalytic performance of Cu‐doped TiO₂, pure TiO₂, and the commercial P25 photocatalyst, a series of controlled experiments were conducted under both full‐spectrum light and UV‐filtered conditions. These experiments aimed to provide a comprehensive comparison of the degradation efficiency of MB, a common dye used as a model pollutant. Each sample was tested in a petri dish containing 10 mL of 50 ppm MB solution. The degradation of MB was monitored over a 240‐min period, with samples collected every 30 min. The collected samples were analyzed using UV–vis spectroscopy to measure the absorbance of MB at its characteristic wavelength, allowing for the calculation of the concentration of MB remaining in the solution. Under visible light conditions (UV‐filter blocked light below 400 nm), the Cu‐doped TiO₂ nanofibers exhibited a significantly higher degradation rate of MB compared to both pure TiO₂ and the commercial P25 photocatalyst. The degradation efficiency was quantified using the C₀/C ratio, where C₀ represents the initial concentration of MB and C represents the concentration at specific time intervals.

As shown in Figure 9b, the Cu‐doped TiO₂ reduced the MB concentration more effectively than the P25 and undoped samples, achieving a degradation efficiency of over 90% within the 240‐min period. This enhanced performance can be attributed to the extended absorption range into the visible spectrum and improved charge separation provided by Cu doping. When tested under full‐spectrum light, which includes both UV and visible light, the Cu‐doped TiO₂ again demonstrated superior photocatalytic activity as shown in Figure 9c. The Figure illustrates that the degradation rate of MB was higher for the Cu‐doped sample compared to pure TiO_2_, and P25 photocatalyst‐ which is known for its high activity under UV light but limited performance under visible light. The doped sample achieved nearly complete degradation of MB within the same time frame, highlighting the effectiveness of Cu doping in enhancing the photocatalytic properties of TiO₂. To further analyze the impact of light spectrum on photocatalytic performance, a direct comparison between the degradation rates under UV‐filtered and full‐spectrum light was conducted, as shown in Figure 9d. The results revealed that while the P25 photocatalyst performed better under full‐spectrum light due to its UV activity, it was still outperformed by the Cu‐doped TiO₂ under both conditions. This indicates that the Cu‐doped sample's activity is primarily driven by its ability to utilize visible light, making it more effective for applications where visible light is the primary light source.

Additionally, the undoped TiO₂ sample, which predominantly consists of the anatase phase, showed limited degradation capability under visible light, achieving less than 20% degradation over 240 min. This was expected given the large bandgap of anatase TiO₂, which restricts its activity to the UV range. However, under full‐spectrum light, the undoped sample showed improved performance but still lagged behind both the Cu‐doped TiO₂ and the P25 photocatalyst, as depicted in Figure 9c.

The quantitative analysis underscores the significant impact of Cu doping on the photocatalytic efficiency of TiO₂. The introduction of Cu^2^⁺ ions into the TiO₂ lattice not only narrows the bandgap, allowing for visible light absorption, but also promotes better charge separation and reduces recombination rates. This combination of factors results in a markedly improved photocatalytic performance, making Cu‐doped TiO₂ a promising candidate for environmental remediation and solar‐driven hydrogen production.

Cu‐doped TiO_2_ shows efficient photocatalytic activity to the degradation of methylene blue due to its improved charge separation ability credited to the presence of the brookite phase. The significant contribution of Cu‐doping into TiO_2_ is to stabilize the brookite phase, and reduce of optical bandgap from 3.2 to 2.63 eV compared to pristine TiO_2_. As a result, the absorption edge of the Cu‐doped nanofibrous mat switches from UV to visible light of the spectrum. This allows to absorb remarkable amount of visible light by the Cu‐doped nanofibrous mat and creates electron and hole pairs. Vequizo et al. reported the presence of moderate electron‐trap depth, compared to anatase and rutile phases, in the brookite phase which reduces the electron–hole recombination process as well as promotes both photocatalytic oxidation and reduction reactions by the material.^[^ ^15a^ ^]^ Thus, the photogenerated electrons and holes have a longer lifetime in Cu‐doped TiO_2_ nanofibrous mat and the ability to contribute to both oxidation and reduction reactions for the degradation of methylene blue. Here, the hole works as a powerful oxidizing agent that converts H_2_O into highly reactive hydroxyl (·OH) radicals. The (·OH) and hole reacts with methylene blue and produces CO_2_ and H_2_O. On the other hand, the negatively charged electrons oxidize the O_2_ and turn into superoxide ions (·O_2_^−^). The ·O_2_^−^ radicals take part in a chain reaction and convert into ·OH radicals, which degrade the methylene blue into CO_2_ and H_2_O.^[^ ^6^, ^20^ ^]^

## 3 Conclusion

In this study, Cu‐doped TiO₂ and pure TiO₂ nanofiber mats were synthesized using a combination of sol‐gel and electrospinning methods and characterized. The key achievements in this study include: 1) the creation of a self‐supported nanostructure with an exceptionally high aspect ratio, enhancing both photo‐absorption and photocatalytic efficiency; 2) the stabilization of the brookite phase, which reduces recombination rates and further boosts photocatalytic performance; 3) a reduction in the bandgap and broadening of light absorption, expanding the range of the photocatalyst's activity; 4) the avoidance of common contaminants such as CuO and Cu₂O, which negatively impact photocatalytic efficiency; and 5) the demonstration of effective dye degradation under natural sunlight alone.

The introduction of Cu as a dopant was intended not only to enhance the photocatalytic properties of TiO₂ under visible light by narrowing its bandgap and improving charge separation efficiency but also to lead to the stabilization of a mixed‐phase structure. A revolutionary observation in this work is the successful stabilization of the brookite phase, which, along with anatase, emerged as a primary structural component in the doped nanofibers. This discovery is significant as brookite is rarely seen in TiO₂ nanostructures and is known for its unique photocatalytic properties. Our comprehensive analyses, including XRD, SEM, TEM, UV–vis spectroscopy, and XPS, provided detailed insights into the structural, morphological, and electronic modifications induced by Cu doping.

The XRD analysis confirmed that both the doped and undoped samples predominantly exhibited the anatase phase, with the doped samples showing additional presence of the brookite phase. The TEM analysis further supported these findings, highlighting the uniform distribution of brookite crystallites in the doped samples, which contributed to their enhanced photocatalytic properties. SEM and morphology analysis revealed that the electrospun nanofibers had diameters ranging from 60–190 nm, with a high aspect ratio beneficial for photocatalytic applications.

The UV–vis analysis demonstrated that Cu doping effectively shifted the absorption spectrum of TiO₂ into the visible light region, with the bandgap reduced from 3.1 eV (pure TiO₂) to 2.62 eV (Cu‐doped TiO₂). This shift was crucial for enabling visible light photocatalysis. The XPS analysis confirmed the successful incorporation of Cu^2^⁺ ions into the TiO₂ lattice, which created electronic states favorable for enhanced photocatalytic activity.

The cyclic voltammetry and chronoamperometry studies showed that the doped TiO₂ samples generated a significant photocurrent under visible light, indicating efficient electron–hole pair generation and separation. The qualitative and quantitative analyses of Methylene Blue degradation further underscored the superior photocatalytic performance of Cu‐doped TiO₂. Under both UV‐filtered and full‐spectrum light, the Cu‐doped samples achieved higher degradation efficiencies compared to pure TiO₂ and commercial P25 photocatalysts.

Overall, the Cu‐doped TiO₂ nanofibers exhibited significantly enhanced photocatalytic activity under visible light, attributed to the combined effects of bandgap narrowing, improved charge separation, and the presence of the brookite phase. These findings suggest that Cu‐doped TiO₂ is a promising material for photocatalytic applications, including environmental remediation and solar‐driven hydrogen production. Future work will focus on optimizing the doping levels and exploring the mechanistic aspects of photocatalysis in greater detail to further improve the efficiency and stability of these materials.

## Conflict of Interest

The authors declare no conflict of interest.

## Supporting information



Supporting Information

Supplemental Movie 1

## Data Availability

The data that support the findings of this study are available from the corresponding author upon reasonable request.
