# Supplementary material for: 3D Self‐Supported Visible Light Photochemical Nanocatalysts
Source: Adv Sci (Weinh). 2025 Mar 24;12(19):2502981. doi: 10.1002/advs.202502981 (PMC12097112; doi:10.1002/advs.202502981)
Supplement: Supplementary file 1 — Supporting Information [file ADVS-12-2502981-s002.docx]

**3D Self-Supported Visible Light Photochemical Nanocatalysts**

*Fateh Mikeili^a^, Mohammad Mahafuzur Rahaman ^a*^, and Pelagia-Irene Gouma^a,b^*

^a^Department of Materials Science and Engineering, The Ohio State University, 140 W. 19^th^ Avenue, Columbus, OH 43210, USA

^b^Department of Mechanical and Aerospace Engineering, The Ohio State University, 201 W. 19^th^ Avenue Columbus, OH 43210, USA

^*^Corresponding author: rahaman.10@osu.edu

**Materials and Methods**

Pure TiO₂ and Cu-doped TiO₂ nanofiber mats were synthesized using a combination of sol-gel and electrospinning methods (blend electrospinning). To prepare the pure TiO₂ precursor, a 10% poly(vinyl pyrrolidone) (PVP) (Supplier: Sigma Aldrich) solution in ethanol (with a molecular weight of 1,300,000 gm/mol) was magnetically stirred for one hour to completely dissolve the PVP as a carrier polymer in the electrospinning process. Separately, Titanium Isopropoxide (TTIP) (Supplier: Aldrich) was diluted in anhydrous ethanol inside a nitrogen glovebox to create a 30% weight per volume solution. This homogeneous precursor solution was then mixed with acetic acid to control and delay the hydrolysis step before being combined with the PVP solution. The final precursor mixture was ultrasonicated until a homogeneous, yellow-colored sol was achieved.

For the Cu-doped precursor, Copper (II) nitrate hemi(pentahydrate) (Sigma Aldrich) was dissolved in ethanol and then mixed with the TTIP solution to achieve a 2% doping level. The resultant sol solutions were immediately transferred into syringe for electrospinning. Using a 22-gauge stainless steel needle as the electrospinning nozzle, the electrospinning parameters were set to a flow rate of 1.6 ml/hour, a working distance of 15 cm, and a voltage of 18 kV. A high-voltage power supply was attached to the needle, with aluminum foil serving as the ground to generate the necessary electric field for the electrospinning process. The collected fibers were subsequently heat-treated in a tube furnace at 500 ˚C for 4 hours to decompose PVP, and further oxidize and crystallize the TiO₂ and Cu-doped TiO₂ samples.

**Characterization and Property Measurements**

The crystal structure and phase composition of the samples were analyzed using a Rigaku Miniflex 6000 X-ray diffractometer, recording XRD patterns in the 2θ range of 20° to 60° with Cu-K_α_ radiation (λ = 1.5406 Å) at 40 kV and 15 mA, a step size of 0.02°, and a scan speed of 2°/min. Raw data for XRD were analyzed using MDI Jade software from CEMAS. Surface morphology and microstructure were examined with a Thermo Fisher Scientific Apreo FESEM in secondary electron (SE) mode, with samples sputter-coated with gold to prevent charging. Elemental composition was determined via energy-dispersive X-ray spectroscopy (EDS) using an EDAX detector on the FESEM setup. High-resolution transmission electron microscopy (HR-TEM) was conducted using a Tecnai 20 microscope at 200 kV, with samples prepared by dispersing material in ethanol and placing a drop onto a carbon-coated copper grid. Surface chemical composition and electronic states were analyzed using a Kratos X-ray photoelectron spectrometer (XPS), recording spectra with monochromatic Al Kα X-rays (energy = 1486.7 eV), a pass energy of 200 eV for survey scans, and 50 eV for high-resolution scans, with charge neutralization and calibration using the C 1s peak at 284.8 eV.

Optical properties were investigated using a UV-Vis spectrophotometer (HR 4000 High-Resolution Spectrometer, Ocean Optics Inc) recording spectra in the 200 to 800 nm range, with samples prepared by dispersing material in a suitable solvent and using a quartz cuvette with a 1 cm path length, performing baseline correction with the solvent as a reference. The photocatalytic activity of the samples was evaluated through the degradation of methylene blue under UV-Vis light. A methylene blue solution (10 mg/L) was prepared, and the initial concentration (C₀) was measured by recording the intensity of the characteristic absorption peak at 665 nm. The photocatalyst was then added, and the solution was irradiated under UV-Vis light, with aliquots taken at 15-minute intervals. The concentration of methylene blue (C) at each time point was determined by measuring the intensity of the 665 nm absorption peak, and the degradation efficiency was calculated using the ratio C/C₀.


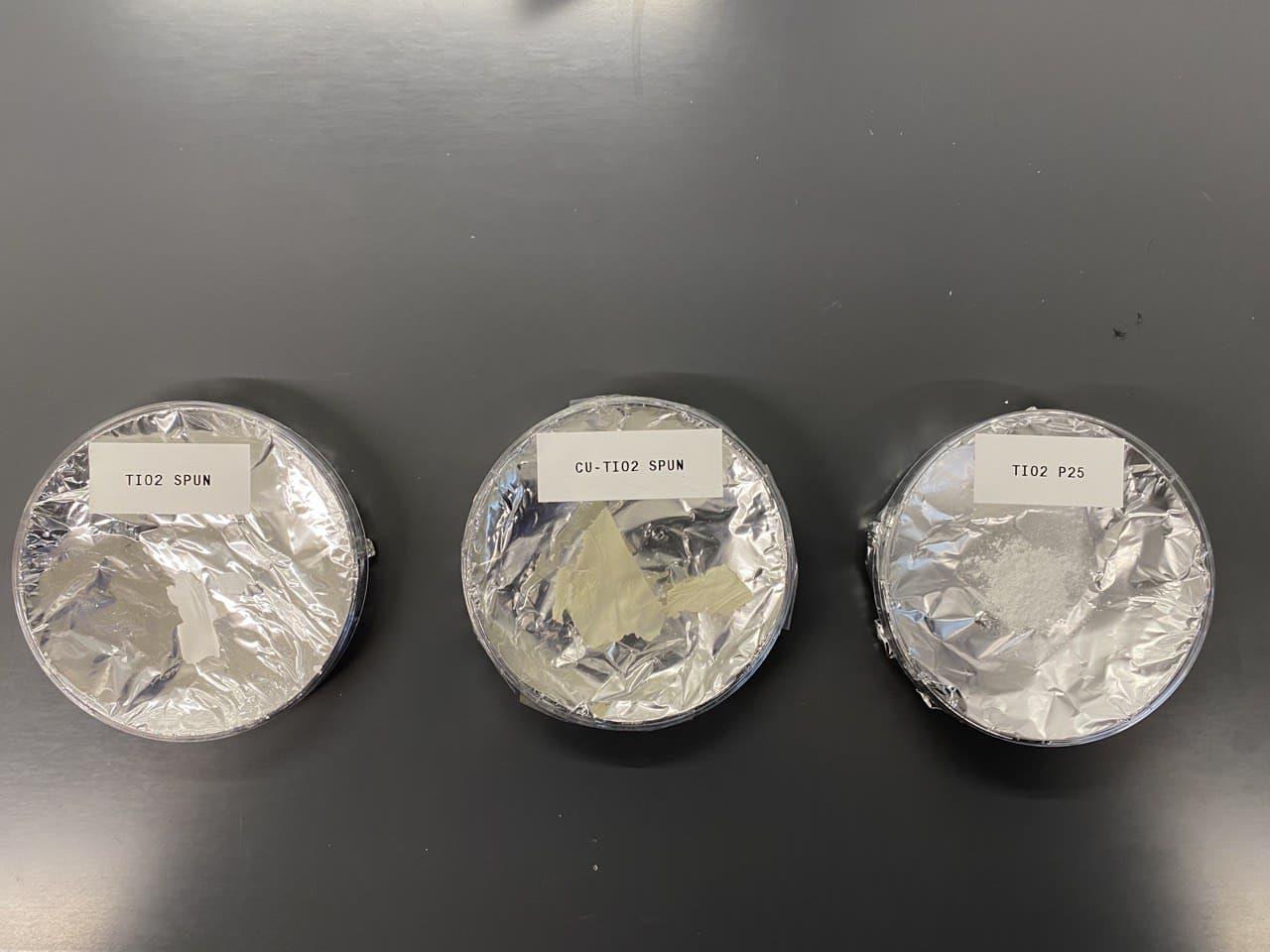


Figure S1: (Left) Pure TiO2 Nanofibrous Mat (Mid) 2% Cu Doped TiO_2_ (Right) Commercial P25 Degussa Nano powder TiO_2_ on aluminum foils


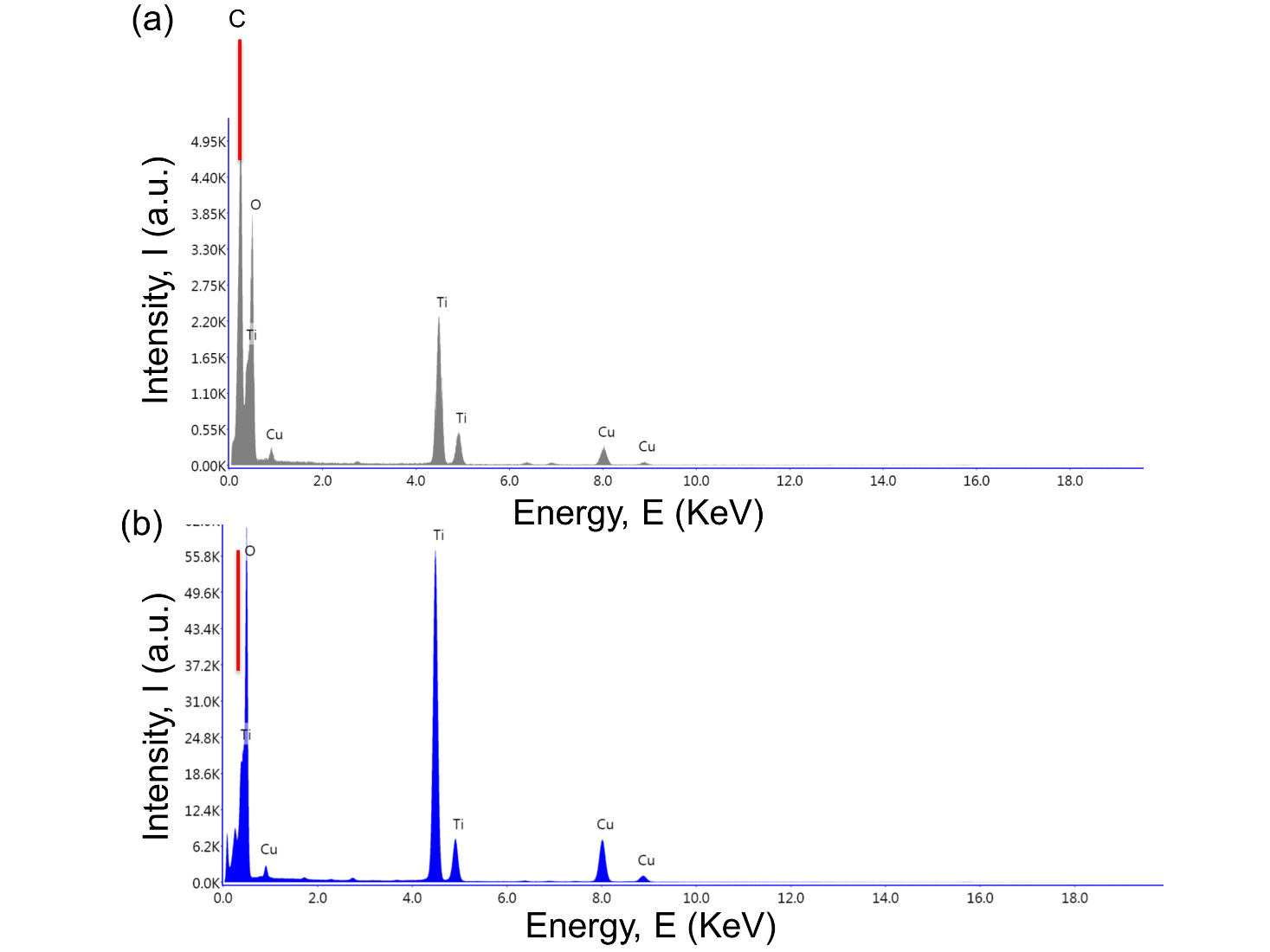


Figure S2: EDX Spectra of (a) as-prepared and (b) heat-treated Cu-doped TiO_2_ nanofibers


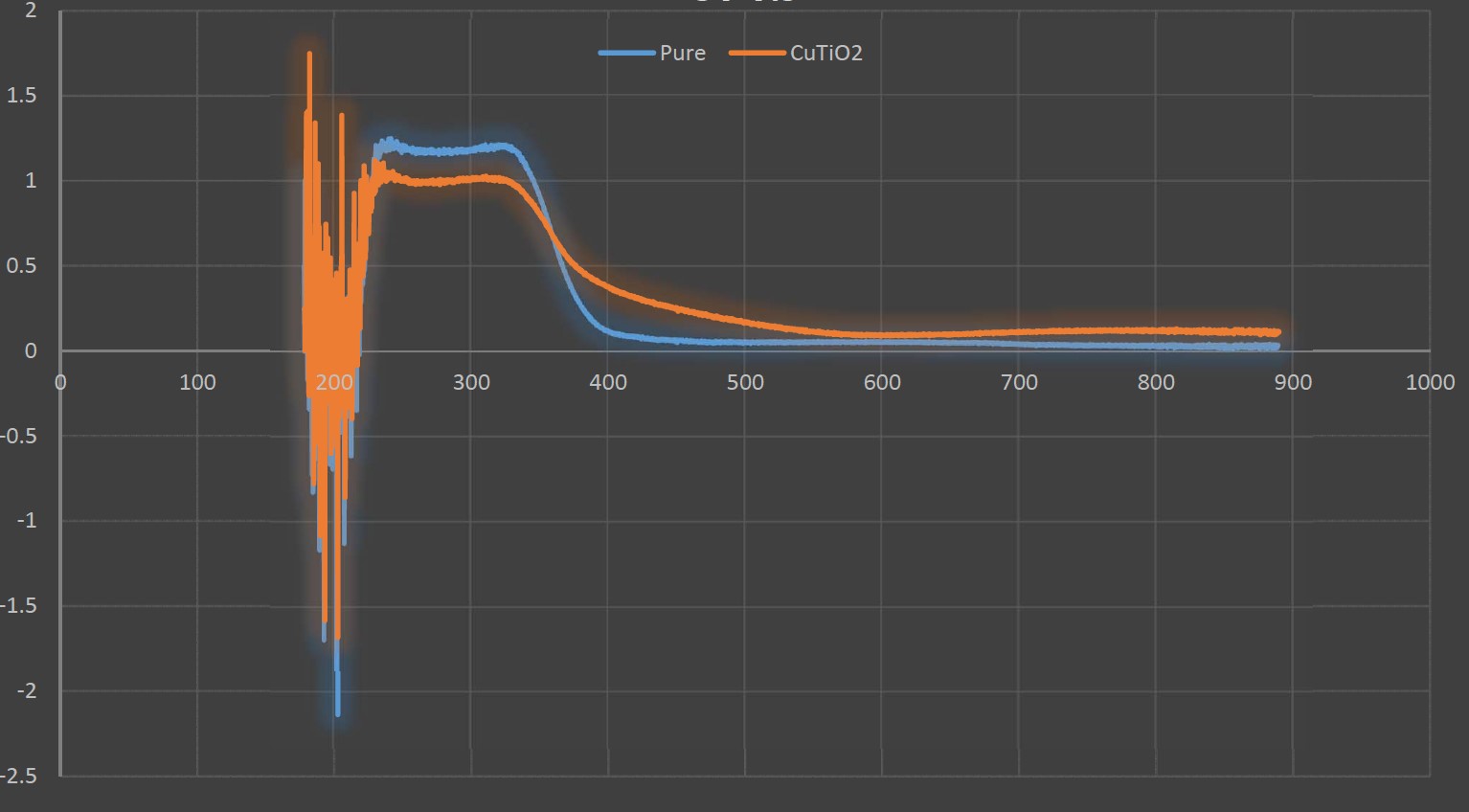


Figure S3: UV – VIS spectra of heat-treated pure and Cu-doped TiO_2_ nanofibers


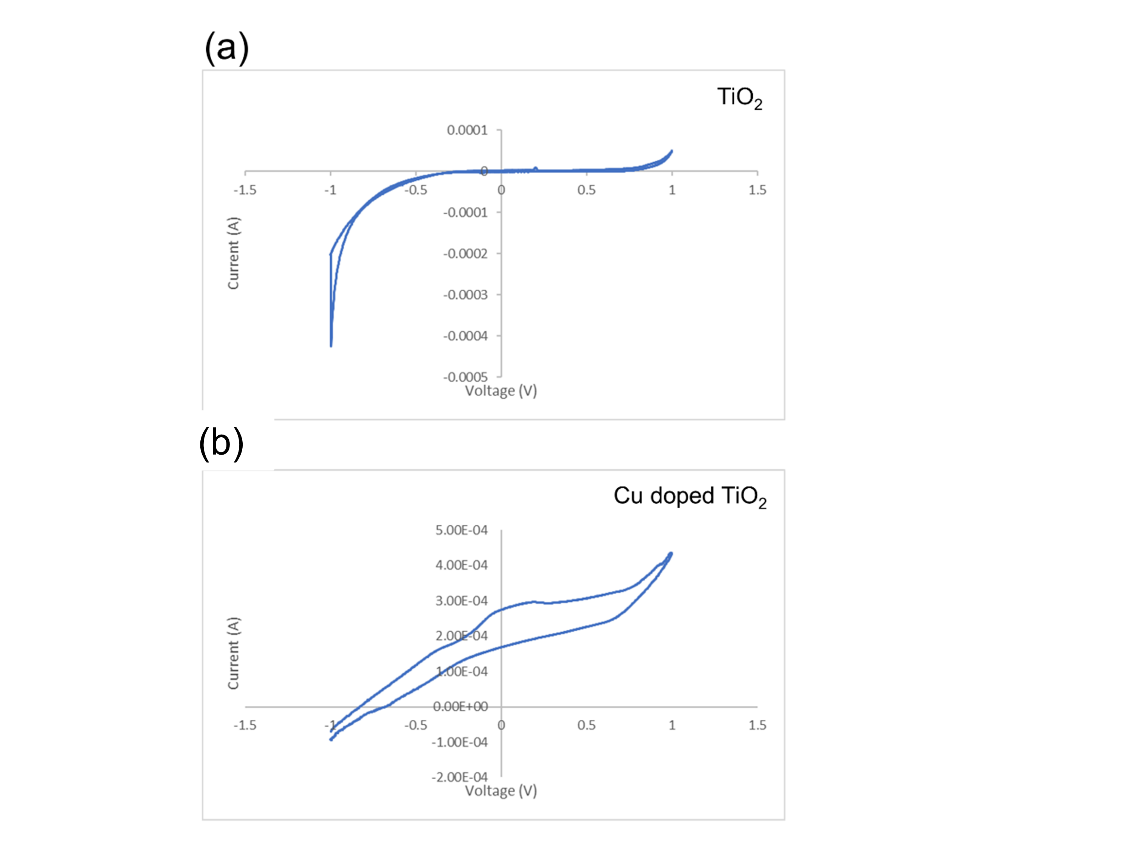


Figure S4: Cyclic voltammetry study of undoped and Cu-doped TiO_2_ within 0.1 KOH solution
